# Interobserver Reproducibility of Diffusion-Weighted MRI in Monitoring Tumor Response to Neoadjuvant Therapy in Esophageal Cancer

**DOI:** 10.1371/journal.pone.0092211

**Published:** 2014-04-04

**Authors:** Robert M. Kwee, Alexander K. Dik, Meindert N. Sosef, Ralph C. M. Berendsen, Sander Sassen, Guido Lammering, Ruud Clarijs, Liekele E. Oostenbrug, Rachel L. G. M. Blom, Roy F. A. Vliegen

**Affiliations:** 1 Department of Radiology, Atrium Medical Center Parkstad, Heerlen, The Netherlands; 2 Department of Radiology, Maastricht University Medical Center, Maastricht, The Netherlands; 3 Department of Surgery, Atrium Medical Center Parkstad, Heerlen, The Netherlands; 4 Surgical Collaborative Network Limburg, Limburg, The Netherlands; 5 Department of Medical Physics, Atrium Medical Center Parkstad, Heerlen, The Netherlands; 6 Department of Radiation Oncology, MediClin Robert-Janker-Clinic, Bonn, Germany; 7 Department of Pathology, Atrium Medical Center Parkstad, Heerlen, The Netherlands; 8 Department of Internal Medicine and Gastroenterology, Atrium Medical Center Parkstad, Heerlen, The Netherlands; 9 Department of Surgery, Academic Medical Center, Amsterdam, The Netherlands; H. Lee Moffitt Cancer Center & Research Institute, United States of America

## Abstract

**Objective:**

To investigate the reproducibility of diffusion-weighted magnetic resonance imaging (DW-MRI) in assessing tumor response early in the course of neoadjuvant chemoradiotherapy in patients with operable esophageal cancer.

**Methods:**

Eleven male patients (mean age 54.8 years) with newly diagnosed esophageal cancer underwent DW-MRI before and 10 days after start of chemoradiotherapy. Reproducibility of apparent diffusion coefficient (ADC) measurements by manual (freehand) and semi-automated volumetric methods was assessed.

**Results:**

Interobserver reproducibility for the assessment of mean tumor ADC by the manual measurement method was good, with an ICC of 0.69 (95% CI, 0.36 to 0.85; *P* = 0.001). Interobserver reproducibility for the assessment of mean tumor ADC by the semi-automated volumetric measurement method was very good, with an ICC of 0.96 (95% CI, 0.91 to 0.98; *P*<0.001).

**Conclusion:**

Semi-automated volumetric ADC measurements have higher reproducibility than manual ADC measurements in assessing tumor response to chemoradiotherapy in patients with esophageal adenocarcinoma.

## Introduction

Esophageal cancer is a disease with a poor prognosis and high mortality. There were an estimated 482,000 new cases and 407,000 patients died of the disease worldwide in 2008 [1]. Preoperative chemoradiotherapy has shown to improve survival compared with surgery alone [2], [3]. However, not all patients benefit from preoperative chemoradiotherapy. In the Chemoradiotherapy for Oesophageal Cancer Followed by Surgery Study trial, as much as 39% of patients had no histopathological tumor regression (tumor regression defined as <10% viable tumor cells in the resected specimen) [3]. Yet, toxicity due to chemotherapy occurs in 11%–90% and there is a risk of radiation-induced complications [3], [4]. In patients who respond insufficiently, inefficient neoadjuvant therapy should be discontinued, and surgery should not be delayed. On the other hand, patients who respond favorably may benefit from additional preoperative treatment and surgery may be delayed or even refrained from. Therefore, there is a need for a method which can differentiate responders from nonresponders early in the course of neoadjuvant treatment. Studies investigating the value of Fluorine 18 (^18^F) fluorodeoxyglucose positron emission tomography (^18^F FDG PET) in assessing response to neoadjuvant treatment show heterogeneous results [5]. Diffusion-weighted magnetic resonance imaging (DW-MRI) may be an attractive alternative to ^18^F FDG PET, because patients do not need to fast before the examination, no exogenous contrast material is required, and acquisition time is much shorter. Therefore, the objective of the present study was to investigate the reproducibility of DW-MRI in assessing tumor response early in the course of neoadjuvant chemoradiotherapy in patients with operable esophageal cancer. In addition, we also investigated the potential of DW-MRI in differentiating responders from nonresponders in a limited number of patients.

## Materials and Methods

### Patient selection

**Figure 1:**
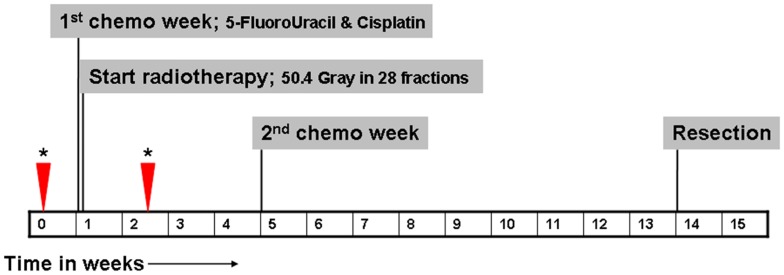
Timing of neoadjuvant therapy, surgery, and MRI (timing of MRI scans is indicated by asterisks and arrowheads).

Patients with newly diagnosed esophageal cancer who were referred to our hospital, which is a regional referral center for the treatment of esophageal cancer, were eligible for inclusion. Only patients who were considered operable and who underwent preoperative chemoradiotherapy were eligible for inclusion. Neoadjuvant therapy regimen and timing are displayed in Figure 1. Patients who already started neoadjuvant therapy before inclusion and patients with standard contraindications for MRI [6] were excluded. This study was approved by the institutional review board of our hospital (Medisch Ethische Toetsingscommissie Atrium-Orbis-Zuyd). All patients gave written informed consent.

### MRI protocol and image review

All MRI scans were performed on one 1.5 Tesla scanner (MAGNETOM Avanto, Siemens AG, Healthcare Sector, Erlangen, Germany). For signal reception, a total of 12 elements of an anteriorly placed body matrix coil and a posteriorly placed spine matrix coil (total imaging matrix system, Siemens AG, Healthcare Sector, Erlangen, Germany), were used. Sagittal and transverse T2-weighted images and DW images were obtained under free breathing so that the entire tumor was imaged. Scan parameters are displayed in Table 1. Total imaging time, including acquisition of scout images, was 10 minutes and 4 seconds. Patients underwent two MRI scans. The first MRI scan was performed within 2 weeks before start of neoadjuvant therapy; the second MRI scan was performed on the 10^th^ day after initiation of neoadjuvant therapy. Time interval between the two scans varied from 12 to 21 days.

**Table 1:** Details of the MRI protocol.

Scan parameters	T2-weighted TSE	T2-weighted TSE	Single-shot SE-EPI DWI with STIR
Imaging plane	Coronal	Transverse	Transverse
TR (ms)	4350	3230	3400
TE (ms)	118	98	78
Turbo factor	13	16	Epifactor 150
Echo spacing (ms)	10.7	10.9	0.7
Bandwidth (Hz/pixel)	201	222	1628
NSA	2	2	6
B-value (s/mm^2^)	NA	NA	0, 300, 1000
No. of slices	26	48	26
FOV (mm)	250	280	380
Matrix size	256	512	192
Slice thickness (mm)	3.0	4.0	4.0
Voxel size (mm)	1.2×1.0×3.0	0.8×0.5×4.0	2.0×2.0×4.0
Acquisition time (minutes)	02:08	04:23	03:14

DWI: diffusion-weighted imaging.

EPI: echo planar imaging.

FOV: field of view.

NA: not applicable.

NSA: number of signal averages.

SE: spin-echo.

STIR: short TI inversion recovery.

TSE: turbo spin-echo.

Quantitative apparent diffusion coefficient (ADC) measurements of the primary esophageal tumor, before and after initiation of neoadjuvant therapy, were performed using a manual and a semi-automated volumetric method, as described below.

**Figure 2:**
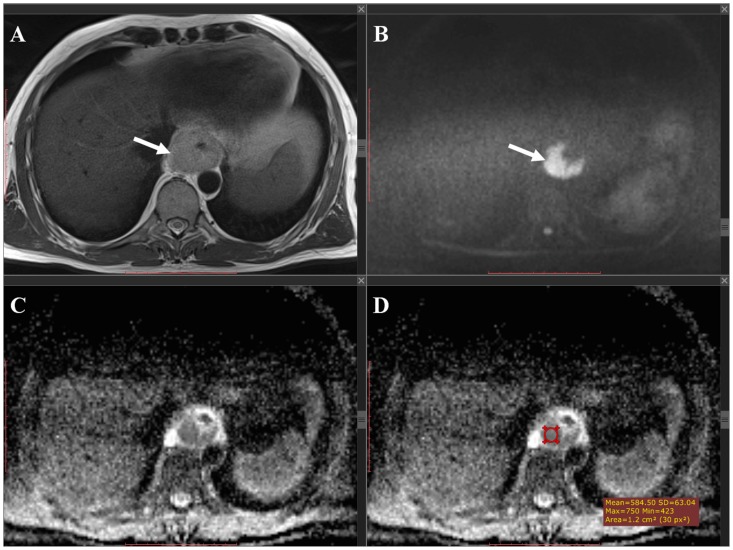
Manual measurement method. T2-weighted (A) and b1000 DW (B) images, and corresponding ADC map (C and D). An esophageal tumor can be depicted as thickening of the esophageal wall (arrow in (A) with high signal intensity on the corresponding b1000 DW image (arrow in B) and low signal intensity on the corresponding ADC map (arrow in C). An ROI was manually drawn in the tumor on the ADC map (red circular region in D) to calculate the tumor ADC value.

For the manual method, measurements were performed on a Picture Archiving and Communications System (Sienet Magic v50 2004, Siemens, Germany). For each tumor, 2 circular regions of interest (ROIs) were manually drawn on the ADC images, which corresponded to the location of the primary tumor on the coregistered transverse T2-weighted and DW images. Subsequently, mean ADC value of the 2 ROIs (corrected for ROI size) was calculated. Figure 2 gives an example. Measurements were performed by a board-certified radiologist with 13 years of experience in cross-sectional imaging (R.F.A.V.), who was blinded to the histopathological results. To assess interobserver reproducibility, measurements were also performed by a fourth-year radiology resident (S.S.) who was blinded to the results of the first reader and to the histopathological results. For further statistical analyses, the average value of the two readers was used.

**Figure 3:**
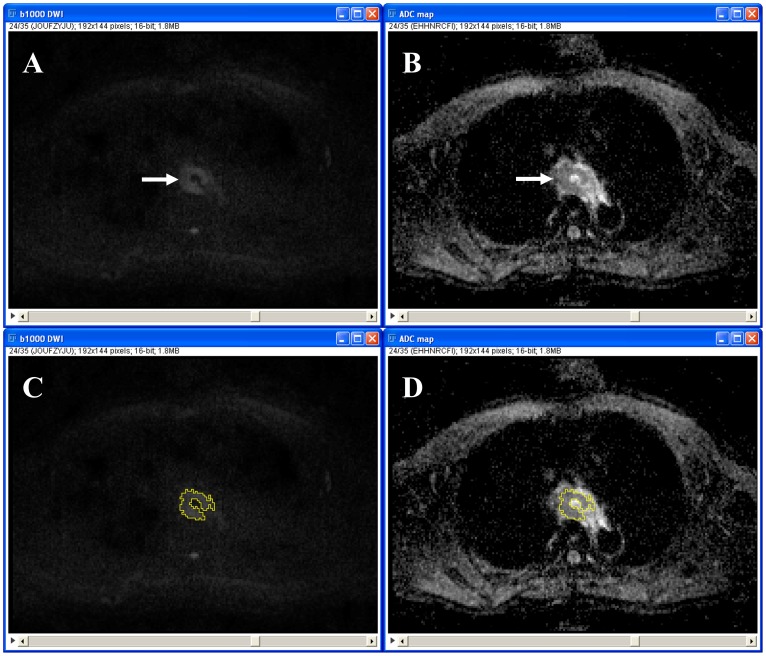
Semi-automated measurement method. B1000 DW image (A and C) and corresponding ADC map (B and D). An esophageal tumor can be depicted as an area of high signal intensity centrally at the b1000 DW image (arrow in A) and as an area of low signal intensity centrally at the corresponding ADC map (arrow in B). After visually selecting a threshold, the software automatically placed an ROI (area bordered by a yellow line in C) around the tumor at the b1000 DW image, which was then copied to the corresponding ADC map (D) to calculate the tumor ADC value.

For the semi-automated volumetric method, measurements were performed using a dedicated open source image processing package (Fiji [7]), based on ImageJ (Rasband, National Institute of Mental Health, Bethesda, USA). The healthy esophagus does not show any high signal on DW-MRI [8]. Therefore, any high signal in the esophagus that exceeded the signal intensity of the surrounding background (lung or air) was considered positive for the presence of tumor. A visually set threshold was applied to the b1000 DW images to generate strictly black and white images, with all the white pixels representing tumor. An image filter called “Particle Analyser” then constructed groups of pixels, i.e. ROIs. Only ROIs which were located around the primary tumor were manually selected and copied to the coregistered ADC maps. ADC values of the ROIs in each slice were assessed, resulting in a volumetric measurement. Mean ADC value of the entire tumor was then calculated. Figure 3 gives an example. Measurements were performed by a fifth-year radiology resident (A.K.D.), who was blinded to the histopathological results. To assess interobserver reproducibility, measurements were also performed by a third-year radiology resident (R.M.K.) who was blinded to the results of the first reader and to the histopathological results. For further statistical analyses, the average value of the two readers was used.

### Histopathological response assessment

The surgical specimens were examined by experienced pathologists and scored according to the tumor regression grade scoring system (TRG) as described by Mandard et al. [9]. TRG was quantified in five grades: TRG 1 (complete regression) showed absence of residual cancer and fibrosis extending through the different layers of the esophageal wall; TRG 2 was characterized by the presence of rare residual cancer cells scattered through the fibrosis; TRG 3 was characterized by an increase in the number of residual cancer cells, but fibrosis still predominated; TRG 4 showed residual cancer outgrowing fibrosis; and TRG 5 was characterized by absence of regressive changes [9]. TRG score was considered the reference standard for tumor response, with TRG scores 1–2 defined as responding and TRG scores 3–5 defined as nonreponding to neoadjuvant chemoradiotherapy.

### Statistical analysis

Statistical analyses were performed by using Statistical Package for the Social Sciences software, version 18.0 (SPSS Inc, Chicago, IL, USA), and MedCalc software (MedCalc, Mariakerke, Belgium). The intraclass correlation coefficient (ICC) was calculated and Bland-Altman plots [10] were constructed to determine interobserver variation in assessing tumor ADC values by the manual and semi-automated volumetric method. Accordingly, agreement in ADC measurements between the manual and semi-automated volumetric method was also determined. ICC values <0.20, 0.21–0.40, 0.41–0.60, 0.61–0.80, and 0.81–1.00 were considered to indicate poor, fair, moderate, good, and very good agreement, respectively. Differences in mean pretreatment ADC and percentage change in ADC between patients with (TRG grade 1–2) and without (TRG grade 3–5) tumor response were assessed by the Mann-Whitney U test and graphically displayed by boxplots. This was done both for the manual and semi-automated volumetric ADC measurements. Correlations between mean pretreatment tumor ADC and TRG score, and correlations between percentage change in mean tumor ADC after start of neoadjuvant therapy and TRG score, were assessed by Pearson rank correlation tests. This was also done both for the manual and semi-automated volumetric measurement methods. Very weak, weak, moderate, strong, and very strong correlation were defined as Pearson ρ's of 0–0.19, 0.20–0.39, 0.40–0.59, 0.60–0.79, and 0.80–1.00, respectively. Scatter plots were constructed to graphically display the correlations. All levels of statistical significance were set at 0.05.

## Results

Between February 2010 and July 2012, 105 patients with newly diagnosed esophageal cancer were referred to our hospital. Sixty of these patients were considered operable, of which 17 were prepared to participate in the present study. All 17 included patients were men, with a mean age of 54.8 years (range 40–71 years). Their characteristics are displayed in Table 2. All tumors were located in the distal third of the esophagus. There were 16 adenocarcinomas and one squamous cell carcinoma. In two adenocarcinomas some degree of signet ring cell differentiation was encountered. Initial clinical tumor stage, based on ^18^F FDG PET/CT and endoscopic ultrasonography findings, varied from cT1N1 to cT4aN3. Of the 17 included patients, three did not undergo the second MRI scan, because of illness during chemoradiotherapy (*n* = 2) and because not showing up (*n* = 1). Two of these patients were not operated on because of death before surgery and because ^18^F FDG PET/CT after neoadjuvant therapy revealed distant metastases. Two other patients were also not operated on because ^18^F FDG PET/CT after neoadjuvant therapy also revealed distant metastases and because liver metastases were detected at the first MRI scan, respectively. In one patient, no tumor was resected because peritoneal metastases were discovered peroperatively. Eventually, there were 11 patients who underwent two preoperative MRI scans (before and during neoadjuvant therapy) and surgical resection of the tumor.

**Table 2:** Tumor type, initial clinical tumor stage, whether or not undergoing 1st/2nd MRI scan, visibility of tumor at DWI scan, results of histopathological analysis, and tumor response to neoadjuvant therapy for all patients.

Patient no.	Tumor type	Initial clinical tumor stage		Patient undergoing 1st/2nd MRI scan	Tumor visible at 1st/2nd DWI scan	Histopathological analysis of resected specimen, TRG score	Tumor response to neoadjuvant therapy (TRG score 1 or 2)
		T	N				
1	Adenocarcinoma	2	1	yes/yes	yes/yes	1	yes
2	Adenocarcinoma	3	1	yes/yes	yes/yes	1	yes
3	Adenocarcinoma	4	2	yes/yes	yes/yes	4	no
4	Adenocarcinoma	2	1	yes/yes	yes/yes	4	no
5	Adenocarcinoma*	3	3	yes/yes	yes/yes	4	no
6	Adenocarcinoma	3	3	yes/yes	yes/yes	4	no
7	Adenocarcinoma	3	1	yes/yes	yes/yes	3	no
8	Squamous cell carcinoma	3	1	yes/yes	yes/yes	1	yes
9	Adenocarcinoma*	3	2	yes/yes	no/no	3	no
10	Adenocarcinoma	2	2	yes/yes	no/no	3	no
11	Adenocarcinoma	2	1	yes/yes	no/no	3	no
12	Adenocarcinoma	3	1	yes/no	yes/NA	2	yes
13	Adenocarcinoma	3	1	yes/no	yes/NA	Death before surgery	
14	Adenocarcinoma	1	1	yes/no	yes/NA	Not operated: scapula metastasis detected at ^18^F FDG PET/CT after neoadjuvant therapy	
15	Adenocarcinoma	4	3	yes/yes	yes/yes	Not operated: liver metastases detected at first MRI scan	
16	Adenocarcinoma	3	0	yes/yes	yes/yes	Not operated: scapula metastasis detected at ^18^F FDG PET/CT after neoadjuvant therapy	
17	Adenocarcinoma	3	1	yes/yes	yes/yes	Peritoneal metastases discovered at surgery; tumor not resected	

*: Some degree of signet ring cell differentiation was encountered.

NA: not applicable.

**Figure 4:**
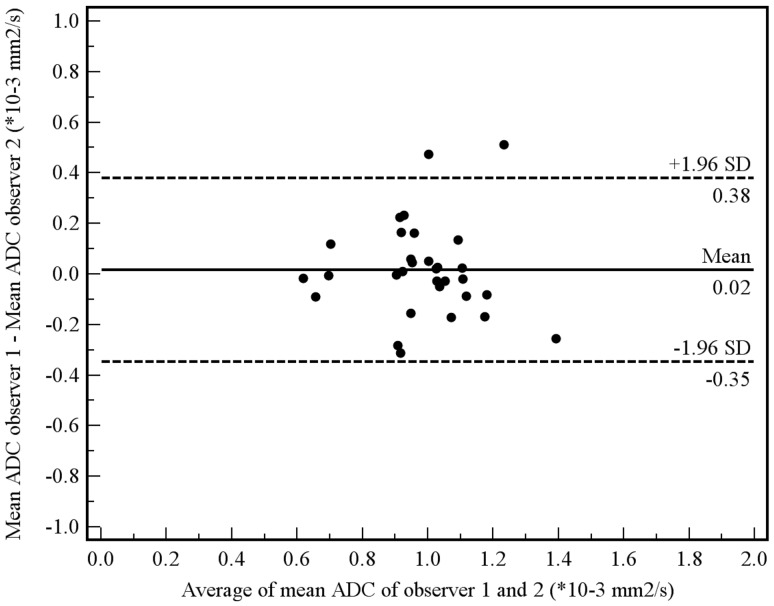
Interobserver reproducibility using the manual measurement method. Measurements from both the first and second MRI scan were included in this analysis. Bland-Altman plot shows the difference between measurements of two observers (R.F.A.V. and S.S.) against the average measurement, with mean absolute difference (continuous line) and 95% CI of the mean difference (dashed lines).

**Figure 5:**
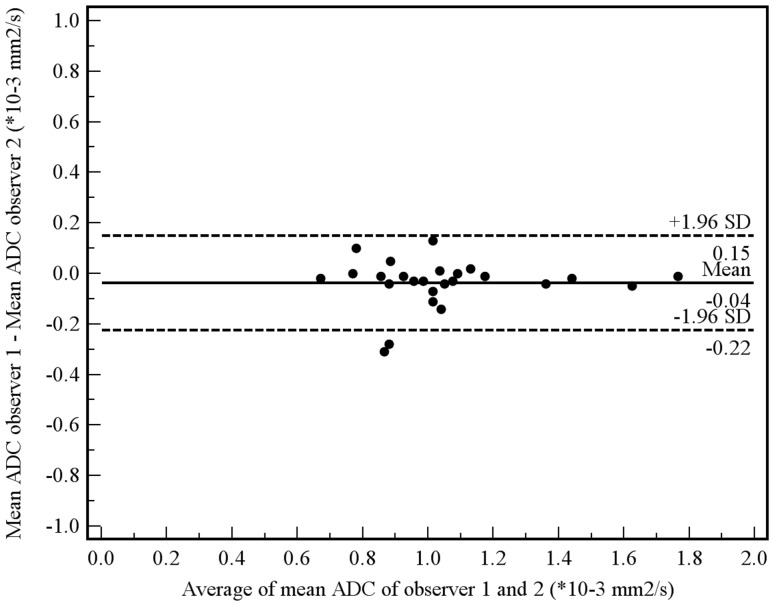
Interobserver reproducibility using the semi-automated measurement method. Measurements from both the first and second MRI scan were included in this analysis. Bland-Altman plot shows the difference between measurements of two observers (A.D.K. and R.M.K.) against the average measurement, with mean absolute difference (continuous line) and 95% CI of the mean difference (dashed lines).

**Figure 6:**
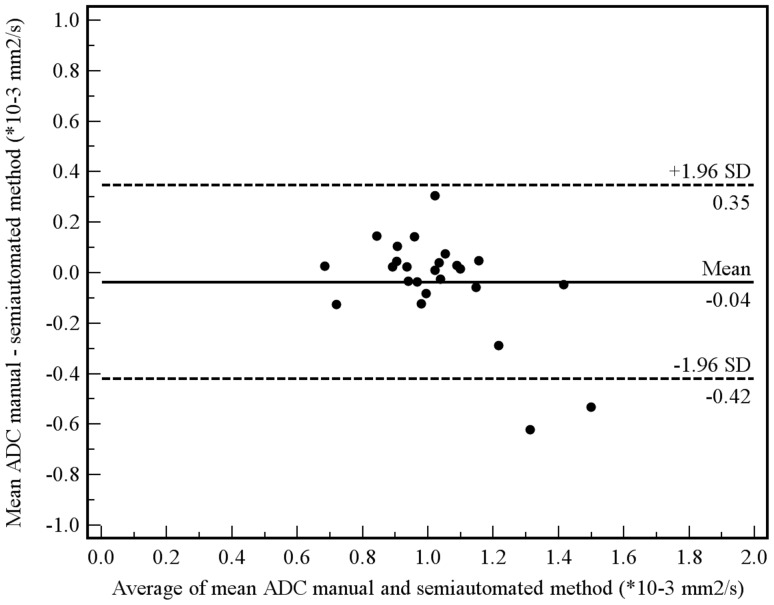
Agreement between the manual and semi-automated method in measuring mean tumor ADC. Measurements from both the first and second MRI scan were included in this analysis. Bland-Altman plot shows the difference between the manual and semi-automated measurements against the average measurement, with mean absolute difference (continuous line) and 95% CI of the mean difference (dashed lines).

**Figure 7:**
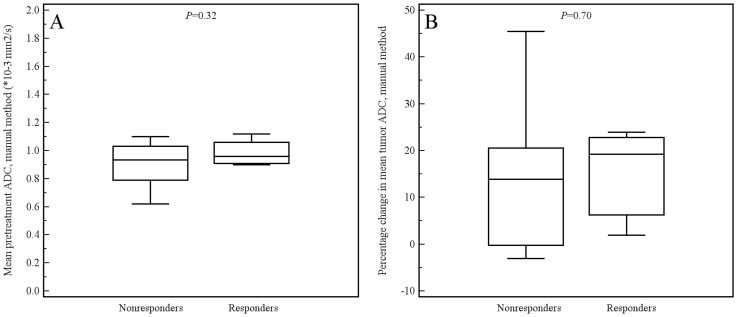
Box plots show the distribution of mean pretreatment ADC values (A) and percentage change in ADC value (B), as measured by the manual method, for patients with and without tumor response.

**Figure 8:**
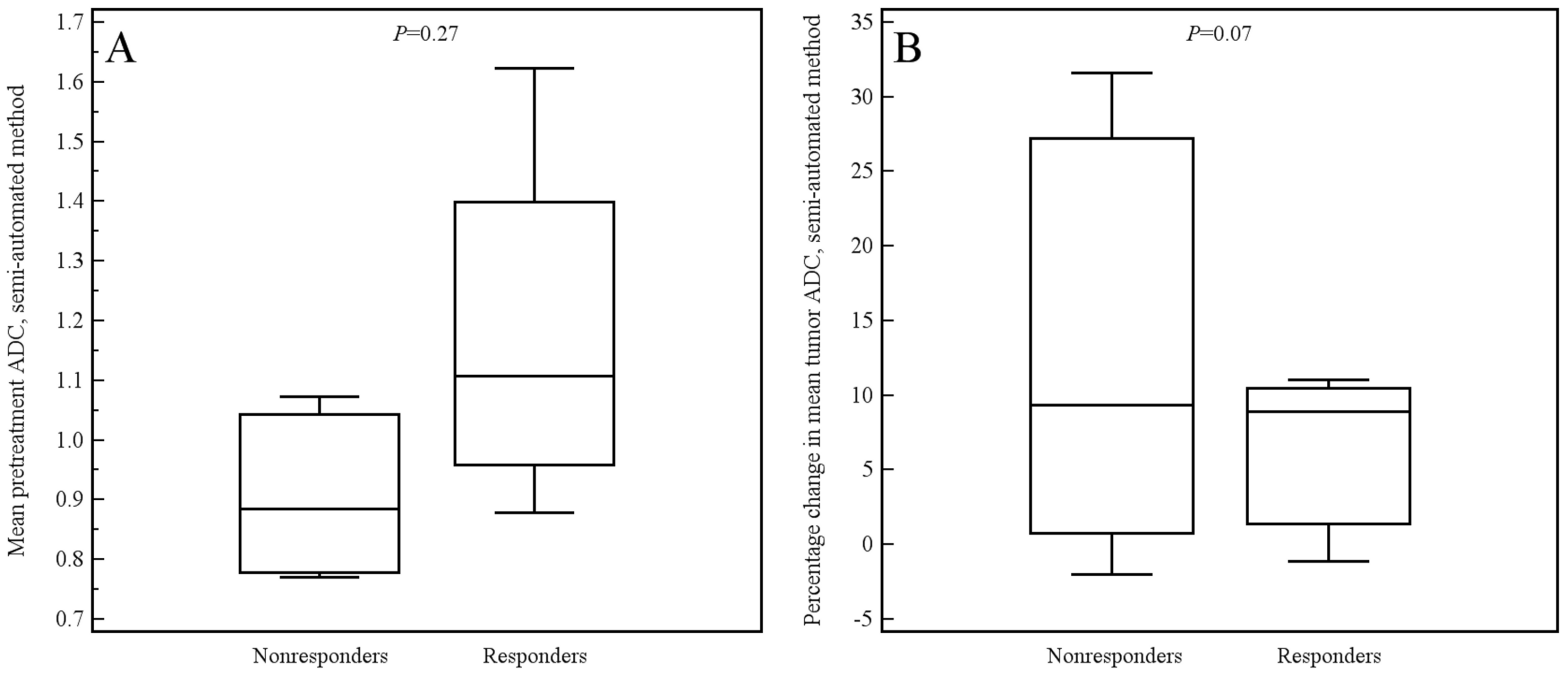
Box plots show the distribution of mean pretreatment ADC values (A) and percentage change in ADC value (B), as measured by the semi-automated method, for patients with and without tumor response.

**Figure 9:**
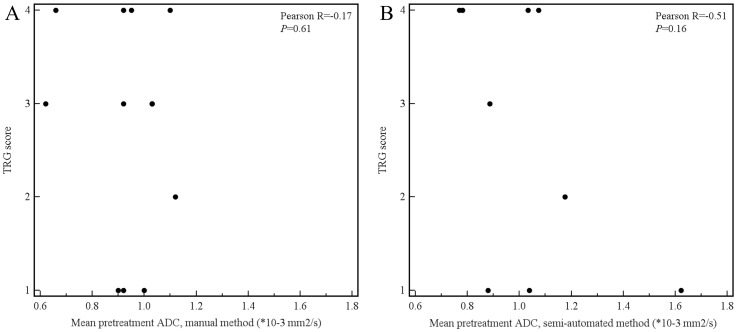
Scatter plots of mean pretreatment tumor ADC value (x-axis) and TRG score (y-axis), both for the manual (A) and semi-automated measurements (B).

**Figure 10:**
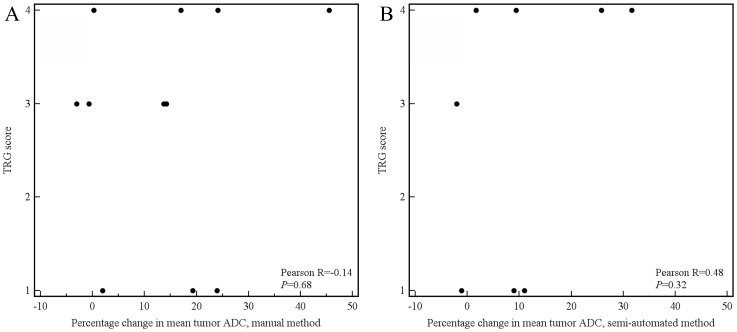
Scatter plots of percentage change in mean tumor ADC value between the first and second MRI scan (x-axis) and TRG score (y-axis), both for the manual (A) and semi-automated measurements (B).

Of the aforementioned 11 patients, 3 had a tumor which was not visible on the DW images. These 3 patients were only included in the manual measurement method, because their tumors could be seen on the coregistered T2-weighted images from which an ROI could be copied to the location of the tumor on the ADC images. Tumor ADC values obtained by manual and semi-automated volumetric measurements before and after start of chemoradiotherapy and percentage change are displayed in Table 3. Interobserver reproducibility for the assessment of mean tumor ADC by the manual measurement method was good (see Figure 4), with an ICC of 0.69 (95% CI, 0.36 to 0.85; *P* = 0.001). Interobserver reproducibility for the assessment of mean tumor ADC by the semi-automated volumetric measurement method was very good (see Figure 5), with an ICC of 0.96 (95% CI, 0.91 to 0.98; *P*<0.001). Agreement between the manual and semi-automated volumetric method for measurement of mean tumor ADC was good (see Figure 6), with an ICC of 0.73 (95% CI, 0.39 to 0.88; *P*<0.001). There were no significant differences in mean pretreatment ADC and ADC change between patients with and without tumor response, neither for the manual (Figure 7A and B) nor for semi-automated volumetric method (Figure 8A and B). We also did not find a correlation between mean pretreatment tumor ADC and TRG score, neither for the manual nor for the semi-automated volumetric measurement method (Figure 9A and B). We also did not find a correlation between change in mean tumor ADC between the first and second MRI scan and TRG score, neither for the manual nor for semi-automated volumetric method (Figure 10A and B). Of note, the interested reader who wishes to see all original data may contact the corresponding author.

**Table 3:** Average and standard deviations (SDs) of mean tumor ADC before and after start of chemoradiotherapy and average percentage change in mean tumor for the manual and semi-automated methods.

	Manual method*	Semi-automated method*
Average and SD of mean ADC value before start of chemoradiotherapy (*10^−3^ mm^2^/s)	0.913±0.170	1.029±0.262
Average and SD of mean ADC value after start of chemoradiotherapy (*10^−3^ mm^2^/s)	1.257±1.015	1.102±0.280
Average percentage change between first and second MRI scan	32.9±15.0	10.7±12.3

* The average value of two readers was used.

## Discussion

As diffusion within tumors is impeded by the presence of cellular membranes and macromolecular structures, treatment with chemotherapy and radiation therapy can result in the loss of cell membrane integrity which can be detected as an increase in mean tumor ADC [11]. DW-MRI for monitoring neoadjuvant therapy has already been applied in a wide variety of cancer types and organ sites, including the liver, breast, bone, soft tissue tumors, cervical tumors, head and neck tumors, as well as rectal cancer [11]. To date, there are only few published studies investigating the clinical value of DW-MRI in evaluating esophageal cancer. An initial study by Sakurada et al. [8] in 24 patients showed that DW-MRI only has a limited role in detecting esophageal cancer and nodal staging. Another study in 123 patients with esophageal squamous cell cancer found that ADC values of primary tumors were lower as clinical T and N stages were more advanced [12]. Aoyagi et al. [13] showed that tumors with lower ADC values had more stromal collagen and higher amount of vascular endothelial growth receptor expression (a marker for tumor neoangiogenesis). A study by the same research group [14], in 80 patients with esophageal squamous cell carcinoma who were treated with chemoradiotherapy, showed that higher mean pretreatment tumor ADC values were associated with longer overall survival. However, the association between change in tumor ADC value after start of chemoradiotherapy and overall survival was not investigated [14]. Sun et al. [15] assessed tumor ADC values before and after start of radiotherapy in 12 patients with esophageal cancer. They showed that patients with a higher increase in tumor ADC value had longer overall survival [15]. However, patients from their study [15] did not receive concurrent chemotherapy and radiotherapy, which is now the standard treatment [2], [3]. In addition, in both Aoyagi et al.'s study [14] and Sun et al.'s study [15], no correlation between ADC measurements and histopathological tumor response or disease-free survival was performed. To our knowledge, our study is the first to perform serial DW-MRI in assessing tumor response to neoadjuvant chemoradiotherapy in patients with operable esophageal cancer.

The deep location of the esophagus, movement related to respiration, peristalsis and cardiac motion, and the presence of local field inhomogeneities caused by susceptibility changes at tissue interface make DW-MRI of esophageal cancer challenging. There are no established protocols for performing DW-MRI of esophageal cancer. The final scanning protocol we used was based on our experience in the use of DW-MRI in other tumors (such as rectal cancer), published literature [8], [12], and test scanning in cooperation with the medical physics department. In three patients of our study (two with initial T2 disease and one with initial T3 disease), the primary tumor could not be detected on the DW-images. This is in conformity with Sakurada et al.'s study [8], where the primary tumor could not be detected in as much as 50.6%. In their study [8], the majority of early esophageal cancers (T1 and T2 tumors) were not detected, while almost all of the T3 and T4 tumors were detected. Thus, it is likely that the low detection rate of DW-MRI is due to the relatively small size of early cancers. Imaging at higher field strength, the use of stronger and faster gradients, and the use of more sophisticated multichannel coils (which enable accelerated parallel imaging) can improve spatial resolution and may increase visibility of smaller esophageal cancers.

We showed that reproducibility of semi-automated volumetric ADC measurements was higher than that of ADC measurements based on the manual placement of ROIs on two slices. This may be expected, as the semi-automated volumetric measurement method leaves less room for variation; the only manual steps to be undertaken are selection of a threshold and selection of ROIs which are within tumor.

In addition, heterogeneity within a tumor may require analysis of the entire tumor volume to obtain reproducible results. Accordingly, a previous study in rectal cancer patients also showed that ADC values obtained from the entire tumor volume were more reproducible than ADC values obtained from an ROI on a single slice or small sample ROIs [16]. Improvements to MRI scanner hardware, as described above, can enhance the resolution of MRI images which may further improve reproducibility of tumor ADC value measurements. A voxel-based analytical method, where changes in individual voxels can be monitored [11], may also improve reproducibility and may provide more reliable results. However, such an approach is challenging, since the esophagus is not a rigid and fixed structure, making spatial registration of DW images obtained before and after start of neoadjuvant therapy difficult.

In the present study, there were 12 and 9 patients for whom pretreatment ADC values (as assessed by the manual and semi-automated volumetric method, respectively) could be correlated to histopathological tumor response. We did not find a significant difference in mean pretreatment tumor ADC between responders and nonresponders. There were 11 and 8 patients for whom change in tumor ADC (as assessed by the manual and semi-automated volumetric method, respectively) could be correlated to histopathological tumor response. As expected, average mean tumor ADC, as measured by both manual and semi-automated methods, increased after neoadjuvant therapy (see Table 3). However, we found no significant difference in change in mean tumor ADC between responders and nonresponders. Although there may truly be no difference, sample size may have been insufficient to detect a significant correlation. Yet, the scatter plots also did not show any trend in relation to TRG score.

The present study has several limitations. First, as already addressed, our study population was relatively small. Most patients who were eligible for inclusion did not want to participate in our study, because they were already facing a difficult time with frequent visits to the hospital and radiation therapy clinic ahead. Because of the low statistical power of the present study, a false-negative result (type II error) cannot be excluded. Further research is needed to confirm or refute our findings. Second, we included mainly patients with adenocarcinoma, which is the most frequent type of esophageal cancer in the Western World [17]. The use of DW-MRI in assessing tumor response to neoadjuvant therapy in patients with squamous cell carcinoma needs to be further investigated. Third, we only performed one MRI scan after start of neoadjuvant therapy. In all patients, this MRI scan was obtained at the 10^th^ day after start of neoadjuvant therapy. This may have been too early or too late to demonstrate significant effect of neoadjuvant treatment. If scanning is performed too early or too late, there may be a decrease in tumor ADC due to either cell swelling or due to the formation of tumor fibrosis, respectively. Future studies need to perform serial DW-MRI scans after start of neoadjuvant therapy to assess whether there is an optimal time point at which change in mean tumor ADC should be assessed. Fourth, we only correlated ADC measurements to histopathological tumor response. The correlation between ADC measurements and clinical endpoints, such as disease-free and overall survival, was not investigated. However, it has been shown that histopathological tumor response after neoadjuvant therapy is strongly related to disease-free and overall survival in patients with esophageal cancer [9], [18]–[20].

In conclusion, the results of the present study show that semi-automated volumetric ADC measurements have higher reproducibility than manual ADC measurements in monitoring response to neoadjuvant chemoradiotherapy in patients with esophageal adenocarcinoma. Our results, although they should be interpreted with care because of small sample size, suggest that pretreatment tumor ADC and change in tumor ADC 10 days after start of chemoradiotherapy do not correlate to tumor response.
